# An Insight on Ellagic Acid Formulations for the Management of Skin Diseases

**DOI:** 10.3390/molecules30234493

**Published:** 2025-11-21

**Authors:** Rebecca Castellacci, Maria Camilla Bergonzi

**Affiliations:** Department of Chemistry “Ugo Schiff”, University of Florence, Via Ugo Schiff 6, 50019 Sesto Fiorentino, FL, Italy; rebecca.castellacci@unifi.it

**Keywords:** ellagic acid, skin application, nanodrug delivery systems, innovative formulations, topical delivery, bioavailability enhancement

## Abstract

The skin is exposed to many environmental stressors, such as UV rays, pollution, and smoke, and psychological stress, which can compromise its structure and function. These factors can cause premature aging, weaken the skin barrier, worsen or induce pathological conditions such as acne and eczema, hyperpigmentation, and melanoma, and slow healing. Ellagic acid (EA) is a polyphenol with various pharmacological effects important for the treatment of skin conditions. It has antioxidant, anti-inflammatory, and depigmenting properties, and it inhibits the enzyme tyrosinase, involved in melanin production, helping reduce dark spots and exhibiting antiproliferative effects against melanoma cells. With its antioxidant effect, it protects the skin against photoaging, combats oxidative stress and signs of aging, such as wrinkles and loss of elasticity, and strengthens collagen and elastin. However, the main limits of EA are its low aqueous solubility, instability, and poor skin permeability that limit its clinical efficacy. This review focuses on EA formulations developed to overcome these limitations and improve its therapeutic effects for skin diseases. Nano-delivery systems such as vesicles, lipidic and polymeric nanoparticles, nanospheres, cyclodextrins, and nanogels have been reported alongside other innovative preparations such as biscuits, sponges, and nanosheets and conventional ones such as ointments, creams, and films.

## 1. Introduction

**Figure 1:**
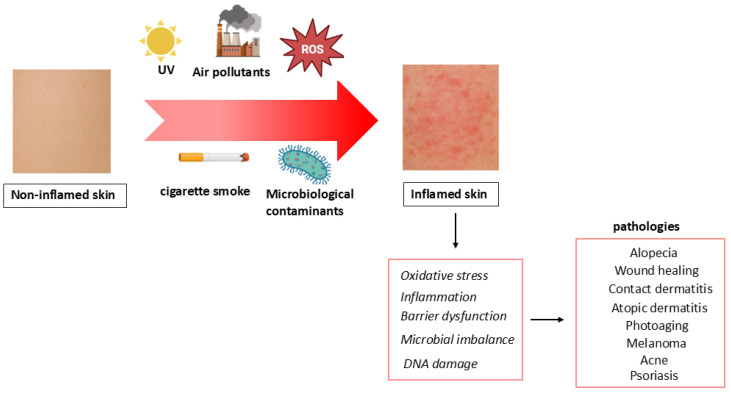
Skin stressors and related skin diseases.

The skin, as the body’s largest and outermost organ, is continuously exposed to a wide range of environmental stressors that can profoundly affect its structure and function (Figure 1). Among the most harmful are ultraviolet (UV) radiation, ozone (O_3_), and cigarette smoke, which is full of pro-oxidant molecules that can trigger significant oxidative damage [1]. Although O_3_ does not penetrate deeply into the skin, it reacts with the outermost layers, depleting antioxidants and compromising the barrier integrity. Similarly, cigarette smoke has been shown to exacerbate oxidative stress, contributing to premature aging and irregular pigmentation. These elements promote the formation of reactive oxygen species (ROS), including superoxide anion (O_2_^−^•), hydrogen peroxide (H_2_O_2_), hydroxyl radical (OH•), singlet oxygen (O_2_) and reactive nitrogen species (RNS), such as nitric oxide (NO). These reactive species quickly destroy the skin’s natural antioxidant defenses, leading to oxidative modifications of lipids, proteins, and DNA. This results in inflammation, impaired barrier function, and long-term tissue damage [2].

This damage is associated with the onset and progression of a wide range of skin disorders like erythema, edema, epidermal hyperplasia, skin aging, contact dermatitis, atopic dermatitis, psoriasis, acne, and alopecia. Moreover, autoimmune skin diseases, such as cutaneous lupus erythematosus, as well as skin cancers (melanoma, basal cell carcinoma, and squamous-cell carcinoma) have been linked to prolonged oxidative stress and inflammation (Figure 1) [3,4].

UV radiation causes oxidative stress through the production of ROS, disrupting the endogenous antioxidative system of the skin and leading to skin inflammation, depigmentation, photoaging, and carcinoma. UV radiation is particularly known for both acute effects (sunburn) and chronic impacts such as photoaging and carcinogenesis through damage to cellular DNA and the dermal matrix. These environmental threats are particularly insidious due to their cumulative and synergistic effects. Repeated exposure accelerates the aging process and increases the risk of chronic dermatological conditions [2].

Air pollutants such as particulate matter, sulfur dioxide, carbon monoxide, polycyclic aromatic hydrocarbons, volatile organic compounds (including toluene and formaldehyde), polychlorinated biphenyls, and even pollen can penetrate the skin barrier, especially when it is already compromised by aging or pre-existing conditions. Once inside, they exacerbate ROS production, damage cellular components, and degrade essential skin proteins like filaggrin and involucrin, increasing skin vulnerability to further environmental insults [5].

In addition to structural damage, pollutants alter the skin’s microbial flora, leading to dysbiosis, a condition recognized as a contributor to inflammatory and immune-mediated skin diseases [6]. Pollutants also activate specific molecular pathways such as the aryl hydrocarbon receptor, which regulates the expression of genes involved in inflammation and immune responses. Persistent activation of these pathways promotes sustained inflammation and has been associated with a wide spectrum of dermatological conditions [7].

The effects of environmental pollutants are often cumulative and synergic, accelerating skin aging and increasing the risk of chronic and degenerative skin disorders over time. Moreover, pollutant-induced oxidative DNA damage, combined with inflammation, plays a key role in carcinogenesis. This complex interplay of oxidative stress, barrier dysfunction, microbial imbalance, and pro-inflammatory signaling provides a mechanistic framework that underscores the impact of environmental stress on skin health [6].

Polyphenols are secondary metabolites of the plants, with high antioxidant properties and protective effects against various diseases related to the free radical damages. Ellagic acid (EA) is a natural polyphenol present in vegetables and fruits such as mangoes, strawberries, blackberries, hazelnuts, walnuts, cranberries, raspberries, muscadine grapes, and almonds. In recent years, it has gained considerable attention due to its pharmacological properties, in particular antioxidant and anti-inflammatory properties that evidence its preventive and therapeutic activity in the treatment of several skin diseases such as melanoma, infections, photoaging, and psoriasis [8]. However, EA has a low solubility and permeability that restricts its clinical application. This review focuses on studies exploring the potential role of EA as an effective agent in the treatment of skin diseases and the formulations proposed for increasing its pharmacokinetic profile and pharmacological activity. Nano-delivery systems such as vesicles, lipidic and polymeric nanoparticles, nanospheres, cyclodextrins, and nanogels have been reported alongside other innovative preparations such as biscuits, sponges, and nanosheets and conventional ones such as ointments, creams, and films.

## 2. Ellagic Acid

**Figure 2:**
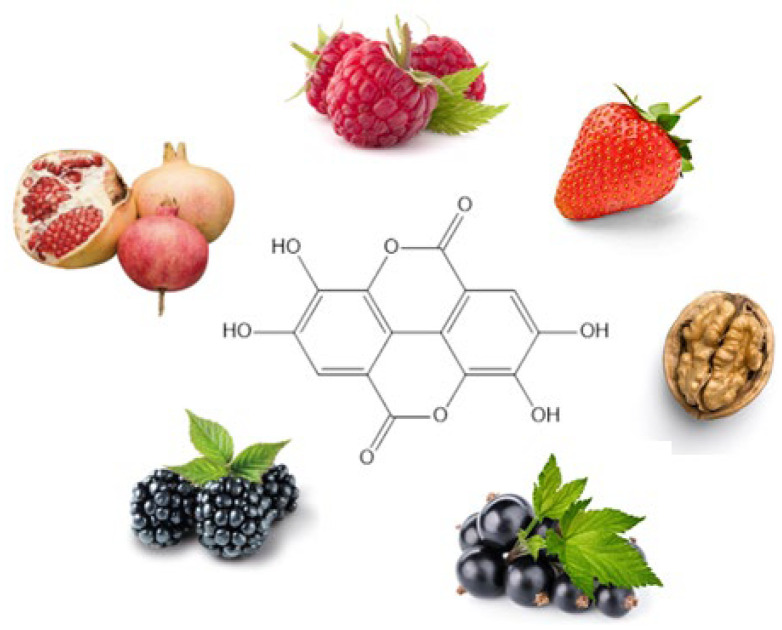
Chemical structure and natural sources of ellagic acid (EA).

EA (Figure 2), discovered by Henri Braconnot in 1831, is a polyphenolic flavonoid with significant pharmacological potential, primarily found in *Punica granatum* L. [9,10]. The plant holds notable cultural value, appearing not only in traditional medical practices but also in religious texts. It is referenced in the Old Testament of the Bible, the Jewish Torah, the Koran, and the Babylonian Talmud, symbolizing fertility, abundance, and good fortune. Beyond its spiritual significance, the pomegranate has been widely used in Ayurvedic and Unani medicine for its antimicrobial, antioxidant, anticancer, and anti-inflammatory properties, as well as its role in regulating lipid and glucose metabolism [11]. In addition to pomegranate, EA is found in a wide range of plant foods. Blueberries contain 120 μg/g (dry weight), strawberries 630 μg/g (dry weight), and notable values are also reported in grapes and dried fruits such as nuts and almonds [12,13]. Among nuts, *Carya illinoinensis* (pecan) is consistently reported as EA-rich (330 μg/g of dry weight) alongside *Juglans nigra* L. (black walnut), containing 590 μg/g of dry weight. Within plants of genus *Robus*, high values are documented for arctic bramble (24.91 mg/g), cloudberry (*R. chamaemorus*, 3151 mg/kg of fresh weight), raspberry (*R. idaeus*, 1500 mg/kg of fresh weight), blackberry (*R. ursinus*, 1500 mg/kg of fresh weight), and boysenberry (*R. ursinus* × *R. idaeus*, up to 4960 mg/kg) [14,15]. Strawberry varieties such as Honeoye, Jonsok, Polka, and Senga Sengana show EA concentrations ranging from 0.12 to 5.64 mg/g [14,16,17]. Reported EA concentrations in ethanolic extracts of longan seed and mango kernel are 1.6 and 1.2 mg/g, respectively [18]. EA has also been identified in extracts from *Vitis rotundifolia* (muscadine grape) cultivars, including the variety Noble (Red), with concentrations of 49.7 mg/kg of dry weight [19].

In addition, fruits from *Rosa* species represent a particularly rich source of EA. A comparative study on fourteen rose species revealed that free EA content in rose hips ranged from 101.1 to 631.3 µg/g of dry weight (mean 208.2 µg/g). Marked interspecies variability was observed, with the highest concentrations detected in *Rosa rugosa*, *Rosa caryophyllacea*, and *Rosa villosa*, whereas lower levels were found in *Rosa tomentosa* and several varieties of *Rosa canina* [20]. Spectrofluorimetric analysis further confirmed the presence of EA in *Rosa canina* species, with concentrations in rose hips ranging from 32.90 µg/g to 44.04 µg/g of the lyophilized forms [21]. In woody species, *Castanea sativa* (sweet chestnut) exhibits substantial tissue-specific differences, with leaves (340–500 mg/kg of dry weight), burs (1410–3210 mg/kg of dry weight), outer shell (240–900 mg/kg of dry weight), and inner shell (~800–1370 mg/kg of dry weight) [22].

Other fruit sources include *Hippophae rhamnoides* (sea *buckthorn* L., 10 mg/kg of dry weight) [23], *Vaccinium* spp. (cranberry, 120 mg/kg of fresh weight) [22], and *Psidium guajava* L. (guava, ~57.2–306 mg/kg of fresh weight) [24]. Among Australian native plants, *Terminalia ferdinandiana* (Kakadu plum) can be exceptionally rich (8796 mg/kg of fresh weight) [15]. Moreover, there is evidence of relatively high levels of EA in several sprouted legumes, such as sprouted *Vigna angularis* (adzuki bean), *Phaseolus vulgaris* (common bean), *Vigna unguiculata* (cowpea), *Pisum sativum* (pea), and *Glycine max* (soybean). Among these, sprouted soybean exhibits the highest concentrations, with values ranging from 45.6 to 48.9 mg/100 g (dry weight) [25].

### 2.1. Extraction Techniques for Ellagic Acid from Natural Sources and Detection Methods

A variety of methods and solvents have been employed to extract EA from natural sources, depending on the plant matrix and target compounds. Among the most common strategies, solid–liquid extraction is a widely used, simple, and effective approach. This method allows the direct contact between the plant material and solvent, allowing the efficient separation and recovery of soluble bioactive compounds without the need for extensive sample pretreatment. Solvent selection is a critical factor, as polar solvents tend to improve the extraction yield of EA. Commonly used solvents are 70% aqueous acetone [26], methanol [16,27], ethyl acetate [28], or 80% aqueous methanol [29,30]. Variants of this method include maceration, stirring [31,32,33], circular shaking [34,35], and the use of a Soxhlet apparatus [36,37].

More recently, ultrasound-assisted extraction has also proven to be highly effective in improving the yield of EA, thanks to its ability to enhance solvent penetration and disrupt plant cell walls. Parameters such as sonication time, power, and bath temperature are crucial to optimize extraction efficiency. It is considered a green technique because it takes less solvent, has a shorter extraction time, and has a simpler operation [38].

Pressurized water extraction has been explored as an environmentally friendly alternative. While it aligns with green chemistry principles, elevated temperatures may compromise the stability of thermolabile compounds as EA. Studies indicate that moderate temperatures (around 40 °C) are preferable, as higher values (e.g., 65–90 °C) can significantly reduce EA yield [39].

Regarding isolation and identification, the most employed analytical method is high-performance liquid chromatography, often coupled with a UV or diode array detector, using a detection wavelength of 254 nm [40,41,42]. Additional techniques include high-performance thin-layer chromatography [43,44], gas chromatography-mass spectrometry [45], and liquid chromatography-mass spectrometry [46,47]. Furthermore, the negative ion mode of electrospray ionization can be effective for EA analysis, with characteristic fragment ions observed at *m*/*z* 284, 257, 229, and 201. Finally, capillary electrophoresis has also been applied for EA analysis [48,49].

### 2.2. Chemistry

EA is a phenolic compound that belongs to the class of hydrolysable tannins, which are divided into gallotannins and the ellagitannins (ETs). EA is derived specifically from the latter. ETs are polyphenols characterized by one or more hexahydroxydiphenoyl (HHDP) groups esterified to a sugar moiety, generally glucose. They exhibit a high degree of structural diversity due to the various ways in which HHDP groups can bind to the glucose core, as well as their tendency to generate dimeric and oligomeric derivatives [50]. ETs are typically unstable both in solution and under environmental conditions, as they tend to undergo hydrolysis and polymerization. Their ester bonds are cleaved when exposed to acidic or basic hydrolysis, leading to the release of HHDP, which spontaneously lactonizes to form EA [51].

Macroscopically, EA appears as a light yellow to brownish powder and has a molecular weight of 302.19 g/mol [9]. Structurally, it is a chromene-dione derivative; it is a dimer of gallic acid, characterized by four fused rings—two of which are lactones—and four hydroxyl groups (Figure 2). According to IUPAC nomenclature, it is also known as 2,3,7,8-tetrahydroxychromeno [5,4,3-cde]chromene-5,10-dione [52]. The -OH groups contribute to its hydrophilic nature, while the two biphenyl rings represent the lipophilic portion. The chemical structure of EA, with both electron-accepting sites (lactone rings) and electron-donating sites (-OH groups), facilitates its role in redox reactions [53], justifying its antioxidant activity.

Unfortunately, EA exhibits two important issues: poor solubility (9.7 μg/mL) [12] and a pharmacokinetic profile marked by limited absorption and a plasma half-life of less than 1 h, as evaluated both in rats and humans [54,55,56].

Improved solubility is observed at increased pH values, up to 33 μg/mL in a phosphate-buffer solution at pH 7.4. Nonetheless, in basic solutions, phenolic compounds are usually converted into quinones through oxidation, losing their stability [10]. EA belongs to class IV of the biopharmaceutical classification system, as it shows both low solubility and low permeability [9]. Furthermore, its high instability in the gastrointestinal medium, along with its potential conversion into urolithins, further limits its bioavailability [57,58].

## 3. EA and Skin

**Figure 3:**
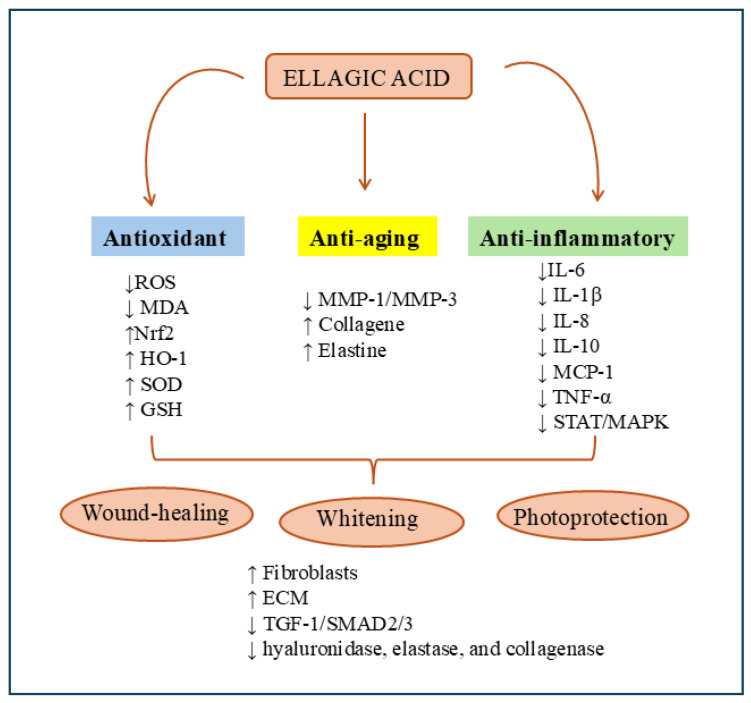
EA skin activity and involved mechanisms. ↓: downregulation; ↑: upregulation.

EA is considered a safe compound for the skin with promising activity as a food supplement and/or in the formulation of skin care products for the prevention or treatment of skin disorders. EA has garnered significant interest in dermatology and cosmetology due to its antioxidant, anti-inflammatory, photoprotective, anti-aging, and anti-melanogenic properties (Figure 3). It helps shield the skin from environmental stress, it minimizes dark spots and wrinkles, and it enhances skin tone and texture. It is also active on psoriasis [59,60]. For that reason, it is a natural source employed in topical formulations and skin care products in the cosmetic field [8].

### 3.1. Anti-Aging and Photoprotection Activity

EA exhibits significant anti-aging and photoprotective properties through multiple and complementary mechanisms. It scavenges free radicals, inhibits matrix metalloproteinases (MMPs) responsible for extracellular matrix (ECM) degradation, enhances the activity of antioxidant enzymes, including superoxide dismutase (SOD) and reduced glutathione (GSH), and activates nuclear factor erythroid 2–related factor (Nrf2)-dependent cytoprotective pathways [8]. Collectively, these effects contribute to maintaining dermal structure and preventing wrinkle formation.

Comparative studies in in vitro and in vivo models consistently reported that EA reduced UV-induced oxidative stress, inflammation, and collagen breakdown. For instance, in UVB-exposed SKH-1 hairless mice, EA treatment reduced wrinkle formation and epidermal thickness while downregulating interleukin-1β (IL-1β) and interleukin-6 (IL-6) [61]. Similarly, in the UVB-irradiated human immortalized keratinocyte cell line (HaCaT), EA reduced the expression of several pro-inflammatory genes such as IL-1β, IL-6, interleukin-8 (IL-8), interleukin-10 (IL-10), monocyte chemoattractant potein-1, and tumor necrosis factor α (TNF-α) [62]. In UVA-irradiated HaCaT cells, EA pretreatment reduced malondialdehyde (MDA) and ROS production, mitochondrial dysfunction, caspase-3 activation, and DNA fragmentation, upregulating Nrf2, heme oxygenase-1 (HO-1), and SOD expression [14].

In addition, EA promoted dermal regeneration by stimulating collagen and elastin synthesis. Aging causes the progressive degradation of elastin fibers, resulting in sagging skin and wrinkles. Duckworth and colleagues proved that EA, alone or combined with retinoic acid, enhanced ECM protein production and improved elastin fiber integrity in human dermal fibroblasts [63]. Such evidence supports EA’s dual role as both a defensive and reparative agent.

Despite abundant in vitro data, clinical validation remains limited. Future investigations should therefore focus on controlled clinical trials using optimized topical delivery systems to confirm EA’s photoprotective efficacy in humans.

### 3.2. Wound Healing and Anti-Fibrotic Activity

The wound healing process is a complex of events that replaces damaged tissue with new cells. The natural response that occurred in the healing process was rapid repair and ensuring the damaged tissues had completely returned to normal conditions. EA modulates multiple aspects of the wound healing cascade, including fibroblast proliferation, ECM remodeling, and inflammatory resolution. It inhibits ECM-degrading enzymes (hyaluronidase, elastase, and collagenase) while upregulating factors that support tissue regeneration, such as basic Fibroblast Growth Factor, Platelet-Derived Growth Factor, and Vascular Endothelial Growth Factor [62,63,64,65,66]. These findings converge toward a pro-reparative profile that prevents excessive fibrosis.

In in vitro models of hypertrophic scar fibroblasts, EA suppressed collagen I and fibronectin expression via inhibition of the TGF-β1 (transforming growth factor β 1)/Smad2/3 signaling pathway [64,65].

In normal human fibroblasts, it upregulated TGF-β receptors (TGFβ-R1/R2) and ECM-related genes such as collagen type I and III (COL1, COL3) and MMP2, indicating its capacity to fine-tune matrix remodeling [66]. The dual modulation, attenuating pathological fibrosis while promoting normal repair, suggests that EA exerts context-dependent regulatory effects.

Animal studies corroborated these findings: 2.5% EA ointment accelerated incision wound closure in male albino rats compared with Betadine^®^ [67].

The combination of EA and carnosic acid, two phenolic compounds, showed synergistic effects on diabetic wound healing and oxidative parameters in diabetic rats [68]. The polyphenols were administered in a rat model of diabetic wound, orally with gavage and topically using a gel of Carbopol 974P. The levels of malondialdehyde, NO, protein carbonyl, oxidation protein products, and MMP 9 decreased, and the rate of wound contraction and collagen levels increased.

EA was revealed to be helpful in the treatment of atopic dermatitis, characterized by impaired skin barrier, skin lesions, and intense pruritus. In TNF-α/interferon-γ-stimulated HaCaT cells, EA inhibited pro-inflammatory cytokine production. The EA application in a mouse model of atopic dermatitis showed improvements in dorsal skin condition, lowered dermatitis scores, reduced transepidermal water loss, and decreased levels of serum Immunoglobulin E, IL-6, and TNF-α [69].

Zhang et al. explored the impact of EA on the aging process of human dermal fibroblast cells. EA delayed senescence by modulating casein kinase II subunit alpha (CSNK2A1), mitigating oxidative stress and inflammation. It upregulated CSNK2A1, decreased β-galactosidase activity, restored cell viability and cycle progression, and reduced apoptosis [70].

Collectively, the evidence indicates that EA not only accelerates wound closure but also improves tissue quality by balancing antioxidant defense, inflammation, and ECM turnover. Nonetheless, these promising outcomes are primarily preclinical; standardized human studies are required to establish clinical translatability.

### 3.3. Melanoma and Hyperpigmentation

Melanoma is a very common skin cancer in people with fair skin. It forms due to an acquired genetic error in melanocytes. Several factors are involved in melanoma, such as cytokines, growth factors, different neuropeptides, hormones, neurotransmitters, biogenic amines, and melatonin. The melanogenesis is triggered by tyrosinase, with a final production of toxic intermediates such as quinones, semiquinones, and ROS.

The presented data underscore EA’s relevance as both a cosmeceutical and a potential adjunct in melanoma prevention, though systematic clinical validation remains necessary.

EA demonstrated a broad spectrum of anti-melanogenic and anti-proliferative effects, acting through diverse molecular pathways. As a copper-chelating agent, it inhibited tyrosinase activity in a dose-dependent, non-competitive manner, effectively reducing melanin synthesis in in vitro and in vivo models. These results were consistent across B16 melanoma cells and brownish guinea pigs, confirming its potential as a natural depigmenting compound [71,72].

Beyond tyrosinase inhibition, EA interfered with oncogenic signaling. The nuclear factor kappa-light-chain-enhancer of activated B cells (NF-κB) pathway dysregulation plays an important role in the melanoma setting, and its inhibition results in an effective strategy against melanoma. Jensen et al. studied the effect of EA on metastatic melanoma cell lines 1205Lu, WM852c, and A375.86. EA showed antiproliferative effects through the promotion of G1 cell cycle arrest. Moreover, the expression of IL-1β and IL-8, as well as the activation and expression of NF-κβ, were decreased in the presence of EA [73].

Moreover, it acted as an allosteric modulator of phosphatase and tensin homolog, restoring its phosphatase activity and inducing G1 arrest via AKT inactivation [74].

In another in vitro study, the treatment of WM115 and A375 cells with EA led to inhibition of the proliferation, migration, and invasion through the Epidermal Growth Factor Receptor (EGFR) signaling pathway. EA decreased p-EGFR and vimentin expression and increased E-cadherin expression in both cell lines [75].

Hyperpigmentation is the most common facial pigmentary disorder. It has been observed that a local increase in melanin synthesis or uneven distribution of melanin can cause local hyperpigmentation or spots. Whitening agents such as hydroquinone, arbutin, and kojic acid are widely used in cosmetics. EA inhibited skin pigmentation resulting from UV irradiation in in vitro and in vivo experiments in brownish guinea pigs [72]. Furthermore, it counteracts oxidation mechanisms similar to those of rhododendrol and other phenolic compounds like raspberry ketone, 4-methoxyphenol, and 4-tert-butylcatechol that can induce melanocyte cytotoxicity. By interfering with this pathway, EA acts as a potential whitening agent and may help prevent hyperpigmentation-related skin disorders [76].

An in vitro study in B16F10 cells demonstrated that EA exerted its anti-pigmentation effects via two synergistic mechanisms: the inhibition of melanin synthesis through autophagy activation in melanocytes and the reduction in oxidative stress through Nrf2-mediated antioxidant responses in keratinocytes [77]. These effects were confirmed using zebrafish models. EA effectively reduced pigmentation and tyrosinase activity, confirming its efficacy in a physiological context.

Ertam et al. evaluated the effect of synthetic EA and plant extracts containing EA on thirty patients with melasma. A decrease in melanin levels after treatment with the EA was observed. In addition, formulations prepared with plant extracts containing 1% EA + 1% plant extract had the same efficacy against melasma as the formulations prepared with synthetic EA (1%) [78].

Kasai performed a double-blind, placebo-controlled trial to evaluate the protective and ameliorative effects of EA-rich pomegranate extract on skin pigmentation after ultraviolet irradiation. Healthy female volunteers were randomly assigned to three groups, namely, high dose (200 mg/d EA), low dose (100 mg/d EA), and control (placebo). Each group received the respective test dose for 4 weeks. EA-rich pomegranate extract, after oral administration, inhibits the effects caused by UV on pigmentation in human skin [71].

### 3.4. Acne

Acne vulgaris is a common chronic inflammatory disorder of the pilosebaceous unit, mainly affecting the face, chest, and back. It is characterized by the appearance of comedones, papules, pustules, and nodules, which can sometimes lead to scarring [79]. The development of acne involves several key factors: excess sebum production stimulated by androgens, follicular hyperkeratinization, leading to pore obstruction, colonization by *Cutibacterium acnes* (formerly *Propionibacterium acnes*), local inflammatory responses, and oxidative stress [80]. Current treatments include topical or systemic retinoids, benzoyl peroxide, antibiotics, and hormonal therapy, but these can cause side effects or lead to resistance. Therefore, there is growing interest in natural compounds with antioxidant, anti-inflammatory, and antimicrobial properties as potential complementary therapies.

Muddathir et al. investigated the anti-acne properties of tannin-related compounds isolated from the methanolic wood extract of *Terminalia laxiflora*. The researchers focused on EA, among other compounds, evaluating its antibacterial activity against *Propionibacterium acnes* and its ability to inhibit lipase. Lipase plays a crucial role in hydrolyzing sebum triglycerides, leading to the release of free fatty acids that can irritate the skin and contribute to acne development. EA demonstrated significant antibacterial activity with a minimum inhibitory concentration (MIC) of 125 µg/mL and a minimum bactericidal concentration of 1 mg/mL. It also inhibited lipase activity with a half maximal inhibitory concentration (IC_50_ = 832.59 µM) and showed strong antioxidant activity in the 2,2-diphenyl-1-picrylhydrazyl (DPPH) assay (IC_50_ = 4.86 µM). Thus, EA possessed multiple activities relevant to acne pathogenesis, including antimicrobial, lipase inhibitory, and antioxidant effects [81].

In vivo testing using a *Caenorhabditis elegans* model confirmed that the combination effectively attenuated biofilm-associated virulence [82].

Furthermore, EA synergized with tetracycline, enhancing biofilm disruption and reducing *Propionibacterium acnes* virulence both in vitro and in vivo in the *Caenorhabditis elegans* model. In fact, in vitro experiments demonstrated that treatment with EA (250 µg/mL) and tetracycline (0.312 µg/mL) reduced biofilm formation by 80–91% without affecting bacterial growth and decreased the production of extracellular polymeric substances by 20–26% [82].

EA was identified as the main active compound in *Castanea crenata* bur extract (CBE) responsible for its biological effects. In vitro studies showed that CBE reduced sebum production induced by palmitic acid in sebocytes by downregulating Sterol Regulatory Element-Binding Protein 1 and Peroxisome Proliferator-Activated Receptor γ. Additionally, CBE suppressed 5α-reductase activity, an enzyme involved in androgen-induced sebum production. The extract also attenuated Toll-like receptor 2 activation in keratinocytes induced by *Cutibacterium acnes* and palmitic acid, thereby blocking NF-κB factor translocation [83].

These findings collectively suggest that EA, either pure or within polyphenolic extracts, acts on multiple pathogenic nodes in acne, combining antibacterial, anti-lipogenic, and anti-inflammatory effects.

### 3.5. Psoriasis

Psoriasis is a chronic, immune-mediated inflammatory skin disease characterized by excessive keratinocyte proliferation and abnormal differentiation. It affects about 2% of the global population, and it is influenced by genetic, environmental, and immunological factors. The most common form, plaque-type psoriasis, presents the red, scaly, and itchy lesions mainly on the elbows, knees, scalp, and lower back. The disease arises from a complex interaction between the immune system and skin cells. Genetic predisposition, particularly variants in the Human Leukocyte Antigen Cw6 gene, combined with infections, stress, or trauma, leads to immune activation. Dendritic cells and T lymphocytes release cytokines like TNF-α, IL-23, and IL-17, which drive inflammation and keratinocyte hyperproliferation [84].

EA showed promising anti-psoriatic properties, mainly by suppressing pro-inflammatory cytokines and improving barrier integrity [59]. Overall, the following evidence indicates that EA can downregulate multiple signaling pathways implicated in psoriasis, including NF-κB and oxidative stress–related cascades. However, clinical validation and formulation optimization are still required before EA can be considered a therapeutic alternative or adjunct to standard treatments.

In HaCaT keratinocytes, EA reduced β-defensin-2 and chemokine (CCL20, CXCL8) expression via inhibition of the NF-κB pathway, while simultaneously enhancing transepithelial electrical resistance, indicating improved barrier function [54].

In vivo, topical treatment with EA ameliorated imiquimod-induced psoriasis in mice, reducing epidermal thickness, scaling, and leukocyte infiltration. Mechanistic studies revealed that EA exerted anti-inflammatory effects by modulating key signaling pathways and decreasing the production of pro-inflammatory cytokines [85].

Comparative analyses of UV-induced psoriasis models further confirmed EA’s anti-proliferative and antioxidant roles. Lakshmi et al. studied the anti-psoriatic effects of EA in a UV-induced psoriasis mouse tail model. Mice were divided into five groups: a disease control group exposed to UV only, a vehicle control group, a standard treatment group treated with 1% salicylic acid ointment, and treatment groups receiving 1% and 2% EA ointments. EA was applied topically daily for three weeks. The treatment significantly reduced epidermal thickness, with the 2% EA group showing an average thickness of 8.0 ± 0.84 mm compared to the disease control group. The anti-psoriatic effects of EA are attributed to its antioxidant properties, which likely inhibit keratinocyte proliferation and reduce inflammation [86].

### 3.6. Androgenic Alopecia

Androgenic alopecia (AGA) is the most common type of hair loss and is a progressive condition that usually starts during adolescence. It is characterized by the gradual shrinking of hair follicles, which leads to thinner, shorter hairs. It can have a significant impact on the mental health and quality of life of affected people. A key factor in AGA is dihydrotestosterone (DHT), a potent form of testosterone. In the hair follicle’s dermal papilla cells (DPCs), the enzyme 5α-reductase converts testosterone into DHT. DHT binds to the androgen receptor, triggering follicle miniaturization and the overgrowth of sebaceous glands, which ultimately transforms thick terminal hairs into fine vellus hair [87,88].

EA exerts a protective effect against androgen-induced hair loss by modulating oxidative stress and ferroptosis-related pathways.

In DHT-treated DPCs, EA prevented ferroptotic cell death, restored mitochondrial function, and activated the Wingless/Int (Wnt)/β-catenin pathway, a key regulator of follicular regeneration. Through this pathway, EA promoted the proliferation and differentiation of DPCs and keratinocytes and the transition of hair follicles from the telogen to the anagen phase, favoring hair regrowth. These findings were confirmed in in vitro models of DHT-treated dermal papilla cells, as well as in in vivo studies using murine models of AGA [89].

While still preliminary, these findings suggest that EA’s antioxidant and anti-ferroptotic actions may complement existing anti-androgenic therapies, providing a multifaceted approach to hair loss management. However, studies are limited, and available data are not easy to compare due to different in vitro models and experimental conditions used.

### 3.7. Antifungal Infections

Superficial fungal infections, caused mainly by *Trichophyton*, *Microsporum*, and *Candida* species, are a widespread dermatological issue. EA exhibits broad-spectrum antifungal activity by targeting fungal membranes and ergosterol biosynthesis. These pathogens can lead to tinea, onychomycosis, and cutaneous candidiasis, often resulting in chronic, recurrent, and uncomfortable lesions. Limitations of current antifungal therapies, including drug resistance, long treatment duration, and poor topical efficacy, emphasize the need for novel, safe, and effective antifungal agents [90]. Taken together, the published studies underscore the potential of EA as a natural antifungal agent and adjuvant to conventional therapies, although dedicated pharmacokinetic and formulation studies are still needed to confirm its safety and efficacy in clinical settings.

Some studies reported EA’s effectiveness against *Candida albicans* (MIC = 25.0 µg/mL), *Candida tropicalis* (MIC 75.0 µg/mL), and *Trichophyton rubrum*, with the latter being the most susceptible (MIC = 18.75 µg/mL). Mechanistic investigations identified inhibition of sterol 14α-demethylase (CYP51) as a primary mechanism. In vivo topical treatments in guinea pig dermatophytosis models confirmed high efficacy, achieving comparable cure rates to terbinafine. EA at 4.0 and 8.0 mg/cm^2^ achieved 75% and 89% cure rates, while 100% was recorded for terbinafine at 2.0 mg/cm^2^ [91]. Similarly, Brighenti et al. showed that EA was effective against *C. albicans* ATCC 18804, with an MIC value of 3.2 µg/mL [92].

Furthermore, EA inhibited *C. albicans* growth and biofilm formation (up to 82% and up to 68%, respectively) by downregulating genes involved in agglutinin-like sequence 3 and in hyphal formation to ~9.1% and ~20.8% of control levels [93].

EA also showed potent activity against fluconazole-resistant *Candida auris*, with very low MIC (0.125–0.25 µg/mL). Mechanistic evidence suggested cell wall disruption as the main target, since the MIC increased up to 128-fold in the presence of sorbitol, a cell wall–protective osmolyte [94]. EA also enhanced fluconazole efficacy, as it reduced the drug MIC up to 32-fold [95].

### 3.8. Potential Risks Associated with the Topical Use of EA

Although EA is generally regarded as safe, some pharmacokinetic and pharmacodynamic interactions have been described after oral administration. Experimental studies indicate that EA can modulate Cytochrome P450 (CYP450) enzymes (mainly CYP1A2, CYP2C9, and CYP2C19) and may increase the bioavailability of CYP2D6 substrates in Wistar rats, such as metoprolol, by inhibiting their metabolism [96,97]. EA has also been shown to enhance the antinociceptive effect of carbamazepine in mice, amplifying both therapeutic and adverse central nervous system effects [98]. Furthermore, clinical sources report that EA may lower blood glucose levels, possibly enhancing the action of antidiabetic agents and leading to hypoglycemia if co-administered [99].

With regard to dermal safety, only one case report described a possible adverse cutaneous reaction to EA [100]. This short communication detailed a case of allergic contact dermatitis in a 33-year-old botanist who had been repeatedly handling for approximately three months *Melianthus comosus* (honey flower), a South African plant naturally containing EA. The patient presented dyspnea and nausea and developed erythematous rashes that resolved within two weeks following topical corticosteroid treatment and avoidance of further exposure. Patch testing confirmed a positive delayed-type hypersensitivity reaction to both the plant extract and purified EA dissolved in petrolatum, identifying EA as the sensitizing compound.

However, this represents a single, isolated case associated with continuous occupational exposure to a botanical source of EA, rather than the pure compound. No additional cases have been reported; thus, EA is not considered a common contact allergen, and these findings support a favorable safety profile of EA for topical use. Nevertheless, further in vivo studies in larger populations would be valuable to confirm its excellent tolerability and to define any potential for hypersensitivity reactions.

## 4. Nanotechnology-Based Delivery Systems for Ellagic Acid in Skin Disorders

**Figure 4:**
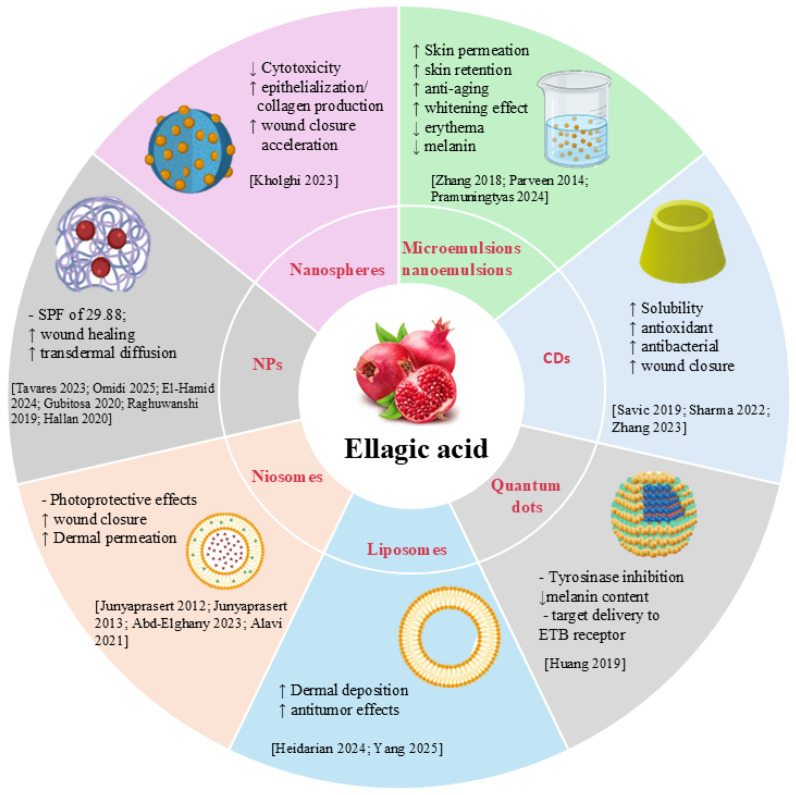
Nanotechnology-based delivery systems for EA skin application [107,112,113,114,115,116,117,118,119,120,121,122,123,124,125,126,127,128,129,130].

The low water solubility, instability, and poor skin permeability of EA limit its clinical efficacy. A proposed solution to these challenges is the encapsulation of EA in nanodrug delivery systems (Figure 4). Nanocarriers have emerged as a promising strategy to improve solubility, stability, and bioactivity and to modulate the release of natural compounds [101,102,103]. Liposomes, polymeric nanoparticles, micro- and nanoemulsions, and lipidic nanoparticles are the innovative formulations explored in the literature for EA delivery [104,105,106,107,108,109,110,111]. This approach has been shown to improve solubility, bioavailability, and pharmacokinetic profiles, leading to an extended potential field of application.

### 4.1. Nanoemulsions and Microemulsions

Nanoemulsions (NEs) using pomegranate seed oil or medium-chain triglyceride oil as the oil phase and loaded with an ethyl acetate fraction obtained from dried pomegranate peel extract have been developed to enhance EA skin permeation to deeper skin layers. The pomegranate peel dried extract is a spray-dried powder standardized to contain at least 40% EA. Skin permeation was evaluated using Franz diffusion cells with porcine ear skin. EA encapsulation efficiency ranged from 70 to 80%, with droplet sizes of 170–220 nm, a polydispersity index (PdI) < 0.2, and a zeta potential around −30 mV. Free EA was undetectable in the receptor compartment, but when loaded into both NEs, it crossed the stratum corneum and was retained in the skin layers [131].

In the cosmetic field, EA was formulated into an O/W NE to improve its skin whitening and anti-aging effects. The NE exhibited an average particle size of 24.28 nm and a zeta potential of −2.93 mV. Its antioxidant and anti-aging activities were assessed using DPPH and tyrosinase inhibition assays, showing performance comparable to vitamin C and superior to free EA. Furthermore, in vivo studies in guinea pig models exposed to D-galactose demonstrated enhanced skin elasticity and a reduction in wrinkle formation [112].

A topical O/W microemulsion (ME) made of polysorbate 80 as a surfactant and propylene glycol as a co-surfactant containing *Punica granatum* extract was evaluated for its effects on skin erythema and melanin levels in 11 healthy Asian volunteers. Its physicochemical characterization revealed an average droplet size of 8.08 ± 0.2 μm with a PdI of 0.362 ± 0.034 and a pH of 5.65 ± 0.3. Skin erythema and melanin levels were assessed at baseline and every 15 days using standardized colorimetry techniques. The active formulation significantly reduced both skin erythema and melanin levels compared to the placebo. These effects are likely attributable to the polyphenolic compounds in pomegranate, particularly EA, which exhibit antioxidant and tyrosinase inhibitory activity [113].

Pramuningtyas et al. [114] focused on the development of nanoemulgels obtained from NEs incorporated into a gel. The formulations contained antimicrobial bioactive compounds extracted from *Punica granatum* peel to combat skin infections caused by *Staphylococcus aureus* and *Staphylococcus epidermidis*. Initially, O/W NEs were prepared using both ethanol and ethyl acetate extracts, showing high EE%, ranging from 98.27 ± 0.11% to 98.77 ± 0.35%, and an average droplet size of approximately 14.20 nm, with a PdI below 0.32. To convert the liquid NEs into semi-solid formulations, they were incorporated into a hydrogel base to obtain the nanoemulgel systems. The hydrogel base consisted of Carbopol 940 (1%) as the gelling agent, NaOH 0.1 N (10%) and triethanolamine for pH adjustment and neutralization, glycerin (5%) as a humectant, methylparaben (0.2%) as a preservative, and distilled water up to 100 mL. The developed nanoemulgels exhibited a pH range of 6.30–6.94, ensuring compatibility with the skin. In vitro antibacterial assays demonstrated that the nanoemulgels effectively inhibited the growth of *S. aureus* and *S. epidermidis*.

In conclusion, NEs and MEs are easy to manufacture and allow versatile incorporation into gels. However, their high surfactant content may raise irritation potential; thus, formulation balance between penetration and tolerability remains crucial. Notably, systematic data on tolerability and safety, particularly after long-term use, are still largely lacking.

### 4.2. Liposomes, Niosomes, and Extracellular Vesicles

EA-loaded LP (EA-LP) (~125 nm, PdI 0.24, zeta potential −12 mV) in combination with liposomes containing doxorubicin (Doxil) exhibited synergistic effects in B16F0 melanoma cells at both 7:1 and 30:1 molar ratios, with increased apoptosis, ROS reduction, and caspase 3/7 activation. In vivo, the combination therapy delayed tumor growth in C57BL/6 mice bearing B16F0 melanoma more effectively than either Doxil or EA-LP alone. This enhanced efficacy may be attributed to the ability of EA to reduce cancer cell resistance to the drug [107].

Deformable vesicular variants such as niosomes further enhance EA permeation through the skin. Junyaprasert et al. developed EA-loaded niosomes composed of mixtures of the non-ionic surfactants Span 60 and Tween 60 in various molar ratios (1:0, 2:1, 1:1, 0:1), combined with cholesterol and Solulan C24. To facilitate the incorporation of EA into the lipid bilayers, solubilizers such as polyethylene glycol (PEG) 400, propylene glycol, or methanol were used. The vesicle size ranged from approximately 124 to 752 nm, and the PdI was below 0.4. The EE% varied between 1.35% and 26.75%, depending on the Span 60/Tween 60 ratio and the type of solubilizer used. Among the tested formulations, those containing PEG 400 achieved the highest EA entrapment efficiency. The formulation with a Span 60:Tween 60 ratio of 2:1 exhibited the best physicochemical stability, maintaining high EA content (95–102%) after 4 months of storage at 4 °C, whereas formulations containing only Tween 60 showed the lowest stability. EA-loaded niosomes enhanced dermal delivery, achieving higher permeation and distribution in both the epidermis and dermis compared to the free EA solution [115].

Subsequently, Junyaprasert et al. explored the impact of chemical penetration enhancers on the skin delivery of EA-loaded niosomes developed in the previous publication. The niosomes were prepared using the reverse-phase evaporation method. The niosomal formulation consisted of Span 60 and Tween 60 in a 2:1 molar ratio, cholesterol as a membrane stabilizer, and Solulan C24 as a steric stabilizer. PEG 400 was included as a solubilizer, while dimethyl sulfoxide (DMSO) or N-methyl-2-pyrrolidone (NMP) was added as a chemical penetration enhancer. EA was incorporated at 1 mol% relative to the total lipid content.

The resulting niosomes were characterized as spherical multilamellar vesicles with mean particle sizes ranging from 312 to 402 nm and a PdI < 0.4. Transmission electron microscopy confirmed the vesicular morphology, while stability studies demonstrated that the formulations remained stable for up to four months at 4 °C. In vitro permeation studies using human epidermis revealed that DMSO-containing formulations significantly enhanced the deposition of EA within the epidermal layers, whereas NMP-based systems facilitated deeper dermal penetration and higher diffusion across the skin. These findings were confirmed by confocal microscopy, which showed that DMSO allowed EA to reach epidermal depths of approximately 30–90 µm, while NMP enabled diffusion into deeper dermal regions (up to 120 µm) [116].

Chitosan-coated EA-niosomes (EE% 74%, size 536 nm, PdI 0.87), prepared with Tween 80 and cholesterol, showed photoprotective effects in HFB4 fibroblasts and protected collagen from UV-induced deterioration. The nanoformulation improved cell survival, upregulated COL1A1 (59.24%), Tissue Inhibitor of Metalloproteinases 3 (Timp3) (83.83%), involved in the enhancement of collagen production and inhibition of protein degradation in the ECM, and Telomerase Reverse Transcriptase (63.61%), involved in protecting cells from telomere shortening; it also downregulated MMP3 by 64.02% with respect to UV exposure [117].

Oligomeric hyaluronic acid (HA)-modified liposomes (EA-HA-L) with HA molecular weights of 0.8, 5, and 52 kDa showed a size < 200 nm, PdI < 0.3, and zeta potential around −5 mV. Employment of HA was attributed to its capacity to be an excellent transdermal enhancer, especially for low molecular weight. EA-HA5k-L enhanced permeation through rabbit skin by 1.28 and 1.65-fold rather than EA-LP and EA solution, respectively. The laser scanning confocal technique assessed EA-HA-L’s ability to pass through the epidermal layer thickness, reaching the dermis. It was facilitated by the reduced E-cadherin expression, the intercellular junction protein that regulates motility and adhesion. HA promoted cellular uptake since it has high affinity for Cluster of Differentiation 44 (CD44) receptors highly expressed in the epidermal keratinocytes and dermal fibroblasts. The formulation showed antioxidant activity against hydroxyl radicals and stimulated collagen and elastin gene expression in a zebrafish model, presenting a cutaneous structure analogous to that of humans [118].

Tian et al. [132] developed a novel approach for diabetic wound healing by encapsulating EA in small extracellular vesicles (sEVs) within a Gelatin Methacryloyl (GelMA) hydrogel. EA was incorporated into sEVs (EA-sEVs) derived from mesenchymal stem cells, which had an average size of 80.25 nm, and then embedded in the hydrogel to allow controlled topical delivery. In vitro, EA-sEVs promoted proliferation, migration, and differentiation of high-glucose-treated human dermal fibroblasts and epidermal keratinocytes through activation of the EGFR. In vivo, in male DB/db mice, the hydrogel accelerated wound closure, improved re-epithelialization, increased collagen deposition, and upregulated EGFR expression at the wound site, mediating the skin repair process. A proniosomal system was designed to encapsulate EPP using a mixture of Span 60 and Tween 20 with cholesterol as a stabilizer. The resulting niosomes displayed nanoscale size (411.3 nm), low PdI (0.266), negative surface charge, and good EE% (68.43 ± 0.24%), remaining stable for several weeks at 4 °C. When tested on 3T3 fibroblasts, the formulation was well tolerated and preserved strong antioxidant activity, suggesting suitability for skin-related applications [119].

Collectively, these findings confirm liposomal and deformable vesicular systems as promising carriers for EA delivery yet emphasize the need for standardized characterization and in vivo clinical validation.

### 4.3. Nanoparticles, Nanospheres, and Quantum Dots

Nanoparticles (NPs) represent another versatile platform for EA encapsulation, enabling controlled release, high drug loading, and tailorable physicochemical properties. Both naturally derived and metal-based NPs have been utilized to enhance EA’s dermal delivery.

EA-zein NPs, showing EE% over 82%, a size of 370 nm, and a spherical shape, exhibited limited cytotoxicity in HaCat and squamous carcinoma (A431) cells. Encapsulation enhanced EA potency in inhibiting hyaluronidase by 77%, lipoxygenase activity by almost 10%, anti-elastase activity from approximately 43% in solution to 92% when loaded in NPs, and collagenase action by 20%. These properties support wound healing, depigmentation, and anti-photoaging benefits. EA showed Sun Protection Factor (SPF) values of 27.41 in solution and 29.88 in NPs. Although 26.20 was recorded for NPs alone, these results suggest its suitability as an organic sunscreen due to the presence of a benzene ring in the structure, characteristic of organic sunscreens. The observed sustained release profile of the formulation (58% after 240 h) was attributed to the interactions between EA’s OH groups and zein’s NH groups [120].

A green nanogel based on arbutin-chitosan NPs (ARB-CHT-NP gel) was developed as a sustainable skin-lightening system. NPs were synthesized via an eco-friendly process, with chitosan as the carrier, and exhibited sizes of 215.7 ± 6.0 nm, positive surface charge, spherical morphology, and high EE% (97.2 ± 0.1%). Ex vivo studies on rat skin showed enhanced transdermal diffusion of ARB from the nanogel compared to ARB gel, highlighting the role of chitosan in improving skin penetration. In vitro tests on B16F10 cells demonstrated superior cytotoxicity, antioxidant, and tyrosinase inhibitory activities of ARB-CHT-NP gel versus ARB-gel, kojic acid, and blank CHT-NPs. In vivo studies in Wistar rats confirmed enhanced skin-lightening effects and good tolerability. These results underline the potential of chitosan-based nanogels to enhance cutaneous absorption and anti-melanogenesis efficacy [121].

Silver nanoparticles (AgNPs) loaded with EPP and HA, presenting a size of about 8 nm, promoted the recovery of cutaneous wounds infected with *Candida albicans*. In vitro studies indicated that the EPP-HA-AgNPs hydrogel exhibited significant antifungal activity against *C. albicans*, as evidenced by reduced biofilm formation and downregulation of biofilm-related genes such as Agglutinin-Like Sequence 1 and Hyphally Regulated 1. Additionally, the hydrogel enhanced fibroblast proliferation and migration, indicating its potential to accelerate wound healing. EPP-HA-AgNPs hydrogel led to over 85% wound closure by day 21, outperforming the commercial ketoconazole cream in a rat model of infected skin wounds. Histopathological analysis revealed improved tissue architecture, increased collagen deposition, and reduced inflammatory cell infiltration. Moreover, molecular analyses indicated modulation of key cytokines and growth factors, including upregulation of IL-10 and downregulation of pro-inflammatory markers such as TNF-α and IL-6, suggesting the promising role of the EPP-HA-AgNPs hydrogel in the treatment of infected cutaneous wounds [122].

A recent study introduced a self-assembled supramolecular system combining green-synthesized gold NPs (AuNPs), chitosan, and EA. The AuNPs were synthesized using *Punica granatum* juice, and chitosan was employed to adsorb EA onto the nanoparticle surface, forming the AuNPs/chitosan/EA complex (mean size 100 ± 40 nm, zeta potential −25 mV). The resulting nanocomposite exhibited multifunctional properties: antioxidant activity with approximately 80% inhibition in the DPPH assay and 60% in the 2,2′-azino-bis(3-ethylbenzothiazoline-6-sulfonic acid) (ABTS) assay, demonstrating significant free radical scavenging; sun protection, achieving an SPF of 20, suggesting effective UV shielding; and tyrosinase inhibition of about 50%, relevant for skin lightening and hyperpigmentation treatment. These findings highlight the potential of this eco-friendly, multifunctional nanoplatform as an alternative to conventional sunscreens and skin-lightening agents, offering the combined benefits of antioxidant protection, UV filtering, and modulation of skin pigmentation [123]. Moreover, green nanotechnology approaches, using biodegradable and biocompatible materials, will be crucial to minimize toxicity and environmental impact.

Raghuwanshi’s study aimed to identify potential inhibitors of heat shock protein 70-1 (HSP70-1) from *Woodfordia fruticosa* to alleviate imiquimod-induced psoriasis-like skin inflammation in a murine model. Using structure-based drug design, bioactive compounds from the ethanolic extract of *W. fruticosa* were screened through molecular docking and molecular dynamics simulations. Among the tested molecules, EA, along with myricetin and quercetin, showed the strongest binding affinities to HSP70-1 and exhibited favorable pharmacokinetic properties and low toxicity. To enhance the bioavailability and therapeutic potential of EA, gold NPs were synthesized, forming EA-loaded NPs (10–20 nm). These NPs were incorporated into a Carbopol 934 gel for topical administration. In an imiquimod-induced murine model of psoriasis, the formulation markedly improved psoriatic lesions, significantly lowering the Disease Activity Index from 5.28 ± 0.19 to 0.63 ± 0.08, reducing epidermal thickness, parakeratosis, keratinocyte hyperproliferation, and serum levels of pro-inflammatory cytokines (TNF-α, IL-22, IL-23) [124].

Two types of EA-loaded NPs were developed using Miglyol + Tristearin and Labrasol + Tristearin for EA dermal delivery (size 180–190 nm, EE% 91–96%). Antioxidant activity was confirmed via DPPH and Ferric Reducing Antioxidant Power (FRAP) assays. Through the FRAP test, activity was lower for both the formulations (60%) than the EA solution (100%). This was attributed to the lag phase before EA solubilization. The 3-(4,5-dimethylthiazol-2-yl)-2,5-diphenyl tetrazolium bromide test showed about 60% viability in EA-NLCs and less than 20% in free EA-treated HaCaT cells [125].

Nanostructured lipid carriers (NLCs) have emerged as valuable alternative drug delivery systems, offering high physical stability and sustained release capabilities. The lipid matrix protects EA from environmental degradation and allows efficient encapsulation of the drug. NLCs loaded with EA-rich pomegranate peel extract (EPP) have been developed for topical applications to enhance the stability and skin penetration of the bioactive compound. The NLCs exhibited a mean size of approximately 200 nm, a PdI of around 0.2, and a zeta potential of −34 mV. The EPP, containing 12% *w*/*w* EA, presented strong anti-tyrosinase activity with an IC_50_ value of 28.54 ± 1.34 µg/mL. When incorporated into NLCs, approximately 90% of the EA present in the extract (i.e., 90% of the 12% *w*/*w* EA) was successfully encapsulated. In vitro release studies demonstrated a prolonged release of the active ingredient over 12 h, following Higuchi’s model. Ex vivo permeation was evaluated using Franz diffusion cells with excised rabbit ear skin, showing that the NLCs significantly enhanced the permeation of EA compared to a control cream containing free extract, indicating improved dermal bioavailability [133].

Dubey and colleagues encapsulated EA into nanosized metalla-cages to enhance its anticancer activity. Photophysical and antioxidant properties were evaluated, showing that specific complexes exhibited high oxygen radical absorbance capacity. In vitro cytotoxicity assays revealed that the EA-loaded metalla-cages inhibited cancer cell growth in the tested human cancer cell lines SK-hep-1 (liver cancer), AGS (gastric cancer), and A549 (lung cancer) by modulating Granulocyte Colony-Stimulating Factor in macrophages [134].

Huang et al. [126] developed transdermal BQ-788/EA@ZnO quantum dots to target melanocytes and inhibit tyrosinase. The ZnO quantum dots (~4 nm in the uncoated form) were functionalized with BQ-788 for selective delivery, as it is an antagonist selectively binding to endothelin receptor type B receptor on the cell membrane of melanocytes. After functionalization, the quantum dots reached a size of approximately 9 nm. The system showed pH-responsive release, with EA released in the acidic environment of the skin. Targeted uptake was observed within 1 h, demonstrating efficient cellular delivery. In vitro, the formulation inhibited tyrosinase activity by 44.2% and reduced melanin content by 37.5% in melanocytes.

In the study by Hussein-Al-Ali et al. [135], EA was immobilized onto functionalized graphene oxide (GO) nanosheets via electrostatic and π–π stacking interactions to form an ELA-GO nanocomposite. The synthesis yields uniformly sized nanoparticles averaging 132 nm in diameter, a PdI of −24 mV, and a drug loading efficiency of 30%. ELA-GO nanocomposite exhibited a sustained release profile over more than 5000 min at physiological pH 7.4, and it was found 98% after 4850 min. Biological evaluation shows that the IC_50_ of the ELA–GO nanocomposite against B16 melanoma cells was 6.16 µg/mL, whereas neither free ELA nor GO produces cytotoxic effects up to 50 µg/mL, highlighting the enhanced anticancer efficacy of the nanocomposite while maintaining safety toward non-cancerous cells (3T3 cells). Finally, the antibacterial activity of the ELA-GO nanocomposite was more evident than ELA alone at the same tested concentration (5 mg/mL) against both Gram-negative and positive bacteria.

A novel polyvinyl alcohol (PVA)-sodium alginate hydrogel was developed by incorporating EA-loaded nanospheres, aiming to enhance wound healing applications. The nanospheres with an average diameter of 241.5 nm and a PdI of 0.129 were dispersed into the hydrogel matrix. The release profile of EA from the hydrogel demonstrated a prolonged release reaching 100% over 48 h. In vitro cytotoxicity was assessed using MTT assays on NIH3T3 fibroblast cells, showing a significant increase in cell proliferation upon treatment with the hydrogel. In vivo wound healing efficacy was evaluated in an animal model (New Zealand rabbits), revealing enhanced epithelialization and collagen production, leading to accelerated wound closure. These findings suggest that the EA nanosphere-incorporated hydrogel is a promising biocompatible dressing for wound healing applications [127].

The effect of surfactant solubilization on the antioxidant activity of EA was investigated to improve its poor aqueous solubility and bioavailability. Span 20 and Tween 60 were added to the aqueous solution of EA and formed stable micelles with an average size of 335.7 nm and a zeta potential of −42.60 mV. Antioxidant activity, assessed by DPPH, ABTS, and FRAP assays, was superior to both free EA and vitamin C, making them suitable for dermatological application. The results demonstrate that surfactant-based systems can effectively optimize the stability, solubility, and bioactivity of EA for pharmaceutical and cosmetic use [136].

### 4.4. Ciclodextrins

Savic investigated the effect of complexation of EA with cyclodextrins (CDs) to improve its solubility and bioactivity. Inclusion complexes were prepared using β-CD and HP-β-CD. Solubility of EA was significantly enhanced, increasing 2.2-fold for the β-CD complex and 2.6-fold for the HP-β-CD complex. Antioxidant activity, measured by the DPPH assay, showed that the IC_50_ of EA decreased from 1.96 μg/cm^3^ to 0.88 μg/cm^3^ for the HP-β-CD complex and 1.27 μg/cm^3^ for the β-CD complex, demonstrating improved antioxidant potency. Antimicrobial activity was maintained against *Candida albicans*, *Proteus vulgaris*, *Klebsiella pneumoniae*, *Escherichia coli*, *Pseudomonas aeruginosa*, *Bacillus cereus*, *Bacillus luteus*, and *Listeria monocytogenes*. Notably, antimicrobial activity against *Staphylococcus aureus* and *Staphylococcus epidermidis* was only detected after complexation with CDs, likely due to the enhanced solubility [128].

Additionally, the cutaneous delivery of EA has been enhanced by forming inclusion complexes (HP-β-CD). In this context, the isolated EA–HP-β-CD inclusion complex was prepared and further incorporated into a gel formulation using carbomer 940 as the gelling agent. Carbomer 940 provided suitable viscosity, spreadability, and bioadhesive properties, ensuring prolonged residence time of the formulation on the skin surface and facilitating drug release and penetration. Structural and physicochemical characterization (FTIR, differential scanning calorimetry, and X-ray diffraction) confirmed the successful encapsulation of EA within the CD cavity, improving dispersibility and protecting the compound against degradation.

In another study, EA was encapsulated into CD-based nanosponges (EA-NS) prepared by both the melt method and microwave-assisted synthesis to improve its pharmaceutical potential. The formulations were characterized for particle size, encapsulation efficiency, solubility, and photostability. Size ranged between 202 and 406 nm, while encapsulation ranged from 46% to 73%. EA solubility passed from 162.5 µg/mL for free EA to 607 and 721 µg/mL for the microwave synthesis and melt method, respectively. Photostability studies conducted on two different aqueous solutions of EA-NS, EANS1 (melt method) and EANS3 (microwave-assisted synthesis), as well as on pure EA, showed that EANS1 was more photostable than both EANS3 and pure EA.

The antioxidant activity of the formulations was assessed, showing that EA-NS retained strong radical-scavenging properties. Among the two preparation methods, microwave-assisted synthesis produced NS with better performance in terms of solubility enhancement and stability [129].

A multifunctional hydrogel was developed by EA-CD inclusion complex into a thiolene photocrosslinkable poly(ethylene glycol) hydrogel. The inclusion complex was formed using mono-(6-mercapto-6-deoxy)-β-cyclodextrin (SH-β-CD) as a crosslinker. Dithiothreitol was added to modulate the hydrogel’s mechanical stiffness, optimizing it for wound dressing applications. The release of EA followed a first-order kinetic release model, maintaining therapeutic concentrations over time for effective wound healing. In vitro studies demonstrated significant antioxidant activity through free radical scavenging, antibacterial activity against *Staphylococcus aureus* and *Escherichia coli*, and anti-inflammatory activity via reduction in pro-inflammatory cytokines. Cytocompatibility assays confirmed excellent fibroblast compatibility, supporting cell proliferation and migration. In vivo, in male rat models with infected wounds, the hydrogel accelerated wound closure, promoted angiogenesis, and enhanced collagen deposition, indicating effective tissue regeneration. In conclusion, the EA–SH-β-CD–thiolene hydrogel combines the therapeutic benefits of EA with a biocompatible hydrogel matrix [130].

Such inclusion-based systems offer simple yet effective means of enhancing EA delivery without the need for complex lipid or polymeric structures. Nonetheless, their limited drug-loading capacity and potential instability in biological fluids warrant further optimization.

### 4.5. Other Formulations

**Figure 5:**
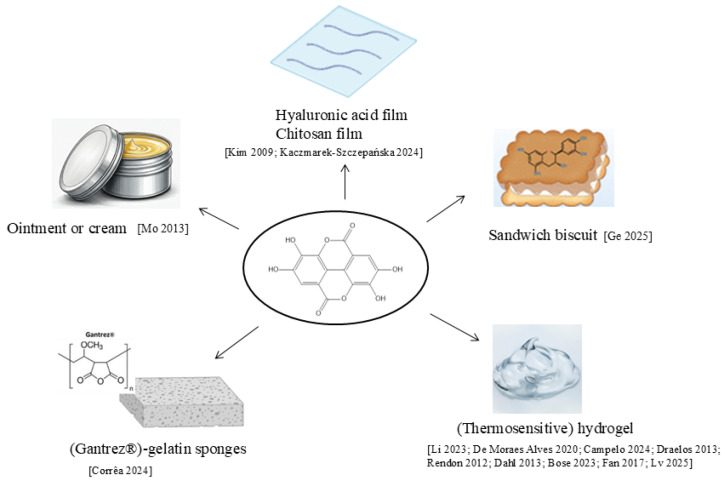
Other formulations developed for EA skin application [58,137,139,140,141,142,143,144,145,146,147,148,149,150].

Other formulations were proposed for EA skin application (Figure 5).

A PEG 400 and PEG 4000 ointment containing 13% *w*/*w* EA pomegranate rind extract improved the amount of accumulated EA in rat skin. The release followed a zero-order kinetic model, with 180.33 ± 18.53 µg/cm^2^ released after 12 h, as measured by Franz diffusion cells [137]. The formulation resulted in promising treatment of a mouse model of contact dermatitis since the effect was comparable to triamcinolone and diclofenac [138].

Chitosan-ellagic acid composite films were developed as a local drug delivery system aimed at inducing apoptotic cell death in human melanoma cells. Chitosan was used as the base matrix, and EA was incorporated at various concentrations (0–1% *w*/*v*). The films were characterized by FTIR, which confirmed the presence of functional groups indicative of successful EA incorporation, X-ray diffraction, which revealed the crystalline nature of EA within the matrix, and scanning electron microscopy, which showed surface morphology and roughness. Contact angle measurements were performed to assess hydrophilicity. Biological evaluation using WM115 human melanoma cells and HS68 fibroblasts demonstrated a dose-dependent antiproliferative effect on melanoma cells, with apoptosis assays confirming induction of apoptotic cell death at higher EA concentrations (0.5% and 1%). Results suggest the developed film’s potential as a local therapeutic system for skin cancer [139].

HA-EA film was prepared by dissolving HA in deionized water at 1% *w*/*v*, while EA was dissolved at 4% *w*/*v* in either 0.0015 M NaOH or 0.1 M acetic acid, depending on the formulation. HA and EA solutions were mixed in different weight ratios (80:20, 50:50, and 20:80), stirred for 1 h at room temperature, and cast onto flat plastic holders to form uniform films. HA-EA film exhibited strong antioxidant and antibacterial activity, particularly against *Staphylococcus aureus*, and was biocompatible with keratinocytes and fibroblasts [140].

A Poloxamer 407-based gel containing amphotericin B and EA was developed for topical application and demonstrated significant efficacy in BALB/c mice infected with *Leishmania major*. Poloxamer 407 is a thermo-responsive triblock copolymer that remains liquid at lower temperatures for easy application and forms a gel at skin temperature, which enhances adhesion and sustained release of the active compounds. In this formulation, EA exhibited significant antileishmanial activity and acted synergistically with amphotericin B [141].

Another Poloxamer 407 thermosensitive gel was used to incorporate EA and gallic acid and evaluated for acute toxicity in *Zophobas morio* larvae. The formulation maintained suitable rheological properties, with no significant changes caused by the incorporation of the polyphenols. Toxicity studies showed high survival rates comparable to controls, indicating the absence of acute toxic effects [142].

The tolerability and skin-lightening ability of EA on the skin were assessed and comparable to a standard formulation of 4% hydroquinone cream and 0.025% tretinoin cream in 82 subjects (7 male, 75 female) ages 25–60 years [143,144]. The results were similar for the EA formulation and the standard cream. Previously, the same efficacy and tolerance were observed in comparing a topical formulation containing EA (0.5%) and salicylic acid (0.1%) versus hydroquinone (4.0%) [145].

EA-loaded poly-methyl vinyl ether-co-maleic anhydride (Gantrez^®^)–gelatin sponges presenting an average thickness between 1.7 mm and 2.7 mm were developed as a novel wound dressing to promote healing and prevent infection. The sponges were prepared by blending Gantrez^®^ with gelatin, followed by homogenization and freeze-drying. Characterization using scanning electron microscopy revealed uniform microporous structures, and the contact angle between 43.7 and 55.5° confirmed the hydrophilic surface. The water absorption capacity of EA-loaded sponges (300–500%) was higher than blank sponges, indicating improved hydration potential for wound application. The release profile of EA was assessed over 120 h, demonstrating sustained release between 30 and 43%, suitable for prolonged therapeutic effect at the wound site. Ex vivo antimicrobial activity was evaluated against *Staphylococcus aureus* and *Pseudomonas aeruginosa* biofilms, showing effective inhibition [146].

Bose et al. [147] investigated the cellular antioxidant and cytotoxic activities of astaxanthin and EA on UV-irradiated SK-Mel-28 skin melanoma cells and developed a gel formulation for topical application using xanthan gum and carboxymethylcellulose as gelling agents. Antioxidant activity, assessed using the 2′,7′-dichlorofluorescein diacetate assay, demonstrated that both compounds significantly reduced ROS in a dose-dependent manner. Cytotoxicity, evaluated by MTT assay, revealed IC_50_ values of 41.39 μg/mL for astaxanthin and 47.23 μg/mL for EA, while EA also increased lipid peroxidation with a median stimulatory concentration of 53.7 μg/mL, suggesting a potential antitumor effect. Thus, their incorporation into a gel formulation could provide a promising strategy for treating radiation-induced skin damage and preventing UV-induced skin cancer. In vitro release experiments demonstrated that EA was liberated in a sustained and controlled manner over 30 h, with an initial burst effect followed by a slower, diffusion-mediated phase consistent with Higuchi-type kinetics. This biphasic profile is particularly advantageous for topical application, as it allows a rapid onset of action together with prolonged availability of the bioactive compound. Transdermal permeation studies using excised rat skin as membranes revealed a significant increase in EA flux compared with the free compound [148].

Zhao et al. developed a zinc ion-coordinated carboxymethyl chitosan (CMCS) hydrogel loaded with EA for accelerated diabetic wound healing. The hydrogel was prepared by crosslinking CMCS with Zn^2+^ ions through coordination bonds and incorporating EA via hydrogen bonding, enabling pH-responsive drug release suitable for the acidic environment of chronic wounds. Characterization using FTIR confirmed the incorporation of Zn^2+^ and EA, while mechanical testing demonstrated structural integrity appropriate for wound dressing. Swelling and degradation studies showed controlled hydrogel behavior, supporting sustained release of EA. In NIH-3 T3 fibroblasts and HUVEC endothelial cells, the hydrogel exhibited strong antioxidant activity, scavenging reactive oxygen species and protecting fibroblasts, thereby promoting cell migration and angiogenesis. It also enhanced re-epithelialization, collagen deposition, and inflammation alleviation to accelerate the wound healing ratio in diabetic mice. The hydrogel modulated macrophage polarization, shifting from a pro-inflammatory M1 phenotype to an anti-inflammatory M2 phenotype, creating a favorable microenvironment for healing. The mechanism of action combines the effects of Zn^2+^, which improves hydrogel mechanical properties and supports cellular functions, with the antioxidant and anti-inflammatory activity of EA, reducing oxidative stress and inflammation at the wound site [58].

In a recent study a double crosslinked hydrogel incorporating zinc-EA metal–organic frameworks (Zn-EA MOFs) combined with mild heat stimulation was developed to accelerate diabetic wound healing. Structural analysis using X-ray diffraction and scanning electron microscopy confirmed uniform integration of Zn-EA MOFs within the hydrogel matrix. Rheological studies, evaluating porosity and swelling ratio, demonstrated suitable mechanical strength for wound dressing applications, while differential scanning calorimetry showed thermal responsiveness compatible with mild heat stimulation. In murine macrophage cell line RAW 264.7, the hydrogel exhibited strong antioxidant activity, scavenging reactive oxygen species and protecting fibroblasts, thereby promoting cell migration and angiogenesis in murine fibroblast cell line L929 and HUVEC cells, respectively. In streptozotocin-induced diabetic rats, the hydrogel enhanced re-epithelialization, collagen deposition, and inflammation alleviation to accelerate the wound healing ratio. Additionally, it modulated macrophage polarization, shifting from a pro-inflammatory M1 phenotype to an anti-inflammatory M2 phenotype. The hydrogel also demonstrated antibacterial activity against common wound pathogens such as *Staphylococcus aureus* and *Escherichia coli*. It was demonstrated both in vitro using the plate coating method and in vivo in bacteria-infected rats. This property contributes to promoting a favorable environment for tissue regeneration [149].

A novel strategy to overcome the poor solubility of EA has recently been proposed through the design of a self-assembled polyphenol–polyphenol “sandwich biscuit” system. In this formulation, EA interacts with gallic acid and catechin via non-covalent forces such as hydrogen bonding, π–π stacking, and hydrophobic interactions, leading to the formation of a stable supramolecular complex with a 180-fold improvement in EA dispersibility in aqueous media. Structural characterization studies elucidated the self-assembly mechanism and confirmed the layered “sandwich-like” organization of the polyphenols. Importantly, this supramolecular system not only enhanced the solubilization and stability of EA but also exhibited synergistic antibacterial activity on *Staphylococcus aureus*, resulting from the combined effects of EA and the co-assembled polyphenols [150].

Overall, these studies confirm the versatility of EA as a bioactive compound in a wide range of topical and transdermal delivery systems, including polymeric films, hydrogels, thermosensitive gels, and supramolecular assemblies. Across different formulations, EA consistently exhibits potent antioxidant, anti-inflammatory, and antimicrobial activities, translating into therapeutic potential for skin disorders such as dermatitis, melanoma, and chronic wounds. The use of polymeric and metal-coordinated matrices, as well as hybrid or supramolecular systems, markedly improves its solubility, stability, and bioavailability. Therefore, more in vivo and clinical studies are still needed to clearly confirm the efficacy and safety of the formulations.

### 4.6. Comparative Evaluation and Challenges

Across different nanosystems, the improvement in EA solubility, stability, and skin permeation is a consistent outcome. Nevertheless, direct comparison among formulations is often hindered by heterogeneity in composition, particle size, experimental models, and evaluation methods. Lipid-based systems generally outperform polymeric and micellar systems in terms of dermal deposition, while hydrogels and nanoemulsions provide superior spreadability and sensory attributes for topical use.

Critical challenges include the lack of standardized evaluation protocols, limited long-term stability data, and the need for toxicity and irritation assessments, particularly for surfactant-rich or synthetic polymer-based systems. Additionally, while numerous in vitro and some animal studies highlight the benefits of nanoencapsulation, clinical evidence remains scarce.

## 5. Conclusions

In vitro and in vivo studies described the numerous pharmacological properties of EA and justified its use in the preparation of products for the prevention or treatment of skin disorders. The main limit of EA is the poor solubility in water, which has a relevant effect on its pharmacokinetic properties. Different new formulations have been developed, and they are effective in improving EA solubility, activity, and bioavailability. These included nanodelivery systems such as vesicles, lipidic and polymeric nanoparticles, nanospheres, cyclodextrins, and nanogels alongside other innovative preparations such as biscuits, sponges, and nanosheets and conventional ones such as ointments, creams, and films. However, despite numerous in vitro studies and fewer in vivo studies, there is a relatively low number of clinical studies, which use different protocols, making the comparison difficult. Furthermore, there is a lack of stability studies and evaluation of key variables for scale-up of the products.

Future research should focus on the rational design of multifunctional and stimuli-responsive EA-based nanocarriers, combining enhanced bioavailability with targeted delivery to specific skin layers or cell types. Greater efforts are also needed to harmonize experimental methodologies, evaluate pharmacokinetics and safety in clinical settings, and explore sustainable, green synthesis approaches for scalable production. Integrating these aspects will be crucial to bridge the gap between preclinical evidence and clinical application, ultimately enabling EA to achieve its full potential as an active ingredient in advanced dermocosmetic and therapeutic formulations.

## Data Availability

The data presented in this study are available on request from the corresponding author.
